# German Anglers’ Views on Global Warming – Implications for Climate Change Monitoring and Management

**DOI:** 10.1007/s00267-025-02291-2

**Published:** 2025-12-01

**Authors:** Wolf-Christian Lewin, Marc Simon Weltersbach, Harry V. Strehlow

**Affiliations:** Thünen Institute of Baltic Sea Fisheries, Rostock, Germany

**Keywords:** Recreational fishery, Environmental perceptions, Global warming, New Environmental Paradigm scale, Aquatic ecosystems

## Abstract

Global warming is affecting aquatic ecosystems worldwide. Recreational anglers could contribute to essential data collection as citizen scientists, serving as a prerequisite for adaptive environmental management. Based on a telephone-diary survey, this study investigated German anglers’ views on climate change impacts on aquatic ecosystems and identified social predictors of these views. The majority of anglers acknowledged the phenomenon of global warming, associating it with extreme weather events, increased aquatic plant growth, and phytoplankton blooms. Only a minority recognised or suspected an impact of global warming on their target fish species. Neither age nor education level significantly influenced anglers’ perceptions of climate warming. Angling motives, gender, angling water, and club membership had little effect, while higher environmental awareness increased the likelihood of recognising climate impacts on aquatic ecosystems. This suggests that environmentally aware anglers may be suitable candidates for environmental monitoring, despite their heterogeneity. The partial inconsistency between anglers’ awareness of climate change and their observed and anticipated future impacts highlights the need for appropriate training as precondition for successfully involving anglers in climate-related environmental monitoring.

## Highlights

- Using a telephone-diary survey, this study assessed German anglers’ potential to monitor climate impacts.
- The vast majority of anglers acknowledged the phenomenon of global warming.
- Anglers recognised warming effects on weather, macrophyte growth and phytoplankton blooms.
- Only few anglers recognised warming impacts on their target fish species.
- Anglers’ environmental awareness makes them potential candidates for monitoring, provided they receive appropriate training.

## Introduction

Global warming is having an increasingly negative impact on aquatic ecosystems (Jeppesen et al., 2012; Schneider et al., 2013; Doney et al., 2012) and it is assumed that these trends will accelerate in the future (Van Vliet et al., 2013; Woolway et al., 2022). Interacting with other anthropogenic stressors, global warming affects phytoplankton and aquatic vegetation (Dhir, 2015; Griffith and Gobler, 2020) as well as the structure of macroinvertebrate and fish communities (Durance and Ormerod, 2007; Jeppesen et al., 2012). The interactions between global warming, eutrophication and habitat changes are likely to contribute to the spread of tolerant species with broad ecological niches, which may lead to substantial biodiversity loss (Markovic et al., 2014; Tanentzap et al., 2020). Adapting to these changes requires monitoring of these trends to detect changes in aquatic ecosystems across different temporal and spatial scales, whether due to climate variability or climate warming (Adrian et al., 2009; Philippart et al., 2011). As long-term data are rare, new baseline monitoring programs are needed because ecosystem changes may occur over increasingly short periods. This makes it crucial to detect early warning signals required for management strategies that identify and prioritize endangered ecosystems for conservation and restoration efforts, and to support adaptation to climate change (Smit et al., 2000; Hughes, 2013).

Millions of people practice recreational angling worldwide in freshwater and coastal areas (Arlinghaus et al., 2020). These people will be affected by climate warming, not least because rising temperatures can lead to harsher weather conditions (Dundas and von Haefen, 2020) and increasing water temperatures, which can affect iconic target fish species (Smialek et al., 2021; Basen et al., 2022). However, warmer climate may also extent the fishing season and make certain target species available in new areas, potentially increasing participation rates in those regions (Hunt et al., 2016; Townhill et al., 2019).

As elsewhere, the warming of lakes in Germany has already been observed over the last few decades (Adrian et al., 2009). Depending on the altitude, latitude and lake type, climate warming influenced stratification as well as ice cover and nutrient budgets. Climate warming favoured hypolimnetic oxygen depletion, early blooms of diatoms, the development of large zooplankton species in spring, and cyanobacterial dominance during summer (Adrian et al., 2006; Livingstone and Adrian, 2009; Wagner and Adrian, 2009; Schwefel et al., 2015). Fish communities are affected in different ways. Souza et al. (2022) showed that dry conditions and elevated water temperature during spring and summer enhanced the viability of carp (*Cyprinus carpio*) populations in Germany. The impacts of climate warming on pike (*Esox lucius*) and pikeperch (*Sander lucioperca*) are not linear, not least because survival, fecundity, and somatic growth rates respond differently to warming water temperatures (Vindenes et al., 2014) which also influence competitive and predatory interactions within aquatic communities (Ohlberger et al., 2011). There are, nonetheless, indications that warmer temperatures might benefit the individual growth rates and population growth of pikeperch, provided that suitable sized prey species are available and water temperatures do not lead to heat stress (Li et al., 2019; Olin et al., 2023). Berggren et al. (2022), for example, showed that warming increased the growth rate of Baltic juvenile pike, while the growth of adult individuals declined and their mortality increased.

In contrast, species with low temperature tolerance, particularly riverine species such as brown trout (*Salmo trutta*) and salmon (*Salmo salar*), are negatively affected by climate warming (Jonsson and Jonsson, 2009; Basen et al., 2022). The decline of brown trout populations in Germany due to habitat loss, changed flow regimes, eutrophication, and river modifications, among other factors, is seen as a warning sign of major climate-related changes in biodiversity in flowing waters (Freyhof et al., 2023). Cold-water fish in the deep lakes might be less affected by climate warming provided that the lakes provide sufficient cold-water refuge (Jeppesen et al., 2012). The western Baltic cod (*Gadus morhua*) which was an important target species for the marine recreational fishery just a few years ago, has collapsed (ICES, 2023) due to past overfishing, and the combination of warming surface water and oxygen deficient deep waters (Receveur et al., 2022). In addition, warming may also promote the immigration and spread of eurytherm and potentially invasive fish species such as round goby (*Neogobius melanostomus*), western tubenose goby (*Proterorhinus semilunaris*), or pumpkinseed (*Lepomis gibbosus*) to name a few (Rabitsch et al., 2013; Worischka et al., 2023; Friedrichs-Manthey et al., 2024).

Citizen science can be a valuable source of information for research and environmental management, particularly when data are collected over large spatial and temporal scales and when motivated volunteers participate (Delaney et al., 2008). Moreover, citizen science can strengthen pro-environmental identities and encourage interest in science and conservation behaviour (Peter et al., 2021). Given the high numbers of anglers and the amount of time they spend at and on the water, anglers could be well integrated into a monitoring system for aquatic ecosystems within a citizen science framework (Pecl et al., 2019; Pita et al., 2020; Tsuboi et al., 2024). Anglers who visit the same water body over a longer period of time might have accumulated ecological knowledge that can help to develop biological monitoring indicators (Shephard et al., 2021), not least as nature experience associated with recreational outdoor activities tends to positively influence environmental concern and stewardship behaviour (Seimer and Knuth, 2001; Church et al., 2025; Van den Heuvel et al., 2025). For example, Graba-Landry et al. (2023) demonstrated that combining scientific data with those from citizen science initiatives (including anglers) could enhance models for quantifying the distribution of fish species at the edge of their range, as well as predicting the availability of habitats in the context of climate warming. Moreover, a recent study showed that angler apps can potentially be used to document spatio-temporal trends in recreational catches of freshwater fishes across the United States. The user-generated data confirmed that climate warming benefited warmwater species while negatively affecting cool- and cold-water fishes (McDonald et al., 2025).

However, anglers are very heterogeneous in terms of socio-demographics, motives for angling, degree of organisation, and, above all, specialisation (Fedler and Ditton, 1994; Arlinghaus et al., 2008). The degree of organisation, i.e. the membership in angling clubs, can have a positive effect on environmental behaviour (Gates et al., 2009). Specialisation encompasses the cognitive (self-assessed skill-level and knowledge), psychological (centrality of the activity to the lifestyle), and behavioural (frequency of participation, avidity) dimensions of commitment to the leisure activity (Ditton et al., 1992; Scott and Scott Shafer, 2001). The level of commitment influences various social settings of anglers’ live including catch and harvest orientation, management preferences, and environmental perceptions (Koemle et al., 2024; Perry et al., 2024; Fisher et al., 2025). Oh and Ditton (2008), for example, showed that the level of conservation concern increased with the specialisation of anglers which was measured using the three dimensions behavior, skill and knowledge and Gray et al. (2015) showed that more specialised anglers possess functional knowledge relating to their main target species that is similar to that of fisheries scientists.

The aim of this study was to investigate whether German anglers observed and expected environmental changes of aquatic ecosystems due to climate warming and which factors (age, gender, education level, centrality, motives for angling, environmental perceptions) would influence this.

First, we hypothesised that highly educated and specialised anglers who were members of angling clubs, were more likely to observe climate-warming-related changes than less specialised anglers. Second, we assumed that a high rating of motives related to the natural environment increases the likelihood of making climate warming-related observations, and that the influence of specialisation on the likelihood of making such observations is mediated by socio-demographics, environmental attitudes, and motives for angling (Navarro et al., 2025). This information could be used to identify groups of anglers who could be recruited to participate in citizen science projects that monitor the impacts of climate warming on aquatic ecosystems.

## Material and Methods

The survey participants were recruited from the general German population using a representative, nationwide telephone screening survey. About 150,000 computer-assisted telephone interviews (CATI) using random digit dialling were carried out from October 2020 to April 2021 to identify anglers in the German population and collect socio-demographic and angling specific data.

Anglers who had fished in German waters in the past 12 months and who were aged 14 or older were asked how many days they had spent angling in 12 months prior to the survey (avidity). They were also invited to participate in a subsequent 1-year diary survey in which they recorded their angling activities in German waters. The diarists were contacted by telephone follow-ups at quarterly intervals during the observation period and asked to rate (i) their skill level, (ii) statements regarding the importance of angling for their quality of life (centrality), (iii) statements regarding their motives to go angling, (iv) statements regarding their environmental perceptions, and finally statements regarding observed and anticipated impacts of climate warming on aquatic ecosystems, angling and future angling behaviour.

The environmental perceptions of the anglers were assessed using the New Environmental Paradigm (NEP) scale (Dunlap et al., 2000). Despite criticism regarding internal consistency and variation in application (Hawcroft and Milfont, 2010), the NEP scale is widely used to predict pro-environmental behaviour (Gatersleben et al., 2014; Berger and Wyss, 2021). The self-assessed angling skill rating ranged from beginner, slightly below average, average, slightly above average, to expert. For the other ratings, a rating scale from 1 (strongly disagree) to 5 (completely agree) was used. The statements included in the survey regarding specialisation (avidity, skill and centrality rating) and motives were based on results of international studies on motivation and specialisation in recreational angling (Fedler and Ditton, 1994; Beardmore et al., 2013). Prior to statistical analyses, the data were weighted to enable generalisation of the conclusions drawn from the analyses (see supplementary material for details on the weighting procedure). The entire survey was conducted by a market and social research company (USUMA GmbH, Berlin, Germany) and planned and supervised by the authors.

Design-based Wald tests and weighted Mann–Whitney U (MW U-tests) tests were used to examine differences in socio-demographic characteristics between anglers who agreed to participate in the diary survey (diarists) and the general angler population identified through the nationwide, representative screening survey. Explanatory factor analysis (EFA) was applied to uncover factors underlying the participants’ motives for angling. The EFA was based on a polychoric correlation matrix to account for the ordinal rating data. Oblimin rotation was used because models that allow the correlation of factors better reflect human behaviour (Costello and Osborne, 2005). The EFA was repeated for two to four dimensions until an optimal solution was reached that incorporated as many statements as possible (factor loadings > 0.4). Statements with factor loadings ≤ 0.4 were removed and not included in the final EFA. The suitability of the data for conducting the EFA was assessed using the Kaiser–Meyer–Olkin criterion (KMO). The appropriate number of factors was determined by parallel analysis (Revelle, 2024), and the goodness-of-fit was evaluated using the Tucker Lewis Index (TLI), the root mean square of residuals (RMSR) and the root mean square error of approximation (RMSEA).

Multinomial regression and ordinal regressions (proportional odds model with logit link) were applied to identify angler-related factors that might have influenced their statements regarding the impacts of climate warming. In the first step, full regression models were built using age, gender, education level, membership in an angling club, avidity (number of angling days in the 12 months preceding the survey), preferred angling water (marine, flowing, standing), mean values of the centrality ratings, self-assessed skill level, and individual factor scores for the four dimensions from the EFA of the motive ratings. The mean values of all NEP statements were used for the regression analysis. The NEP scale can be treated as a single variable (Dunlap et al., 2000; Amburgey and Thoman, 2012) because the focus of this study was on the relationship between environmental perceptions and the perceived impacts of climate change, rather than on specific dimensions of environmental perceptions. Prior to the regression analyses, negatively worded statements of the NEP scale were reverse-coded, after which mean values were calculated and Cronbach’s alpha was calculated to test for internal consistency of the NEP-related statements. We used the mean values of rating scale responses instead of medians, as we assumed the distances between ratings (e.g., from strong decrease to strong increase) were consistent for individual anglers across all responses. In the second step, insignificant variables were manually removed from the regression models as long as their removal decreased the Akaike information criterion (AIC). Model fits were assessed by likelihood ratio tests, comparing the null models to the final models, calculating Nagelkerke pseudo *r*², and conducting Hosmer and Lemeshow as well as Lipsitz goodness-of-fit tests. The Brant test was used to assess the proportional odds assumption for ordinal logistic regression (Fagerland and Hosmer, 2017). Before running the regressions, the variance inflation factor was calculated to test for collinearity among the variables.

Kruskal–Wallis tests, followed by Wilcoxon pairwise tests and false discovery rate adjustment, were used to investigate whether anglers who targeted carp (*C. carpio*), pike (*E. lucius*), pikeperch (*S. lucioperca*), cod (*G. morhua*) and salmonids (mainly anadromous brown trout (*S. trutta*)) differ in their statements about climate impacts.

The software R 4.3.2 (R Core Team, 2023) and the R packages ‘survey’ (Lumley, 2004), ‘psych’ (Revelle, 2023), ‘Likert’ (Bryer and Speerschneider, 2016) and ‘ordinal’ (Christensen, 2023) were used to conduct the statistical analyses and visualise the rating scales.

## Results

### Number of Anglers in Germany and Participation

The total number of anglers in Germany was estimated at 1,676,200 (1,629,500–1,724,200 95% confidence interval) of which about 80% fished in inland and 20% in marine waters. Based on 144,451 complete telephone interviews, 5781 households with anglers were identified and 2793 interviews with anglers conducted. A total of 1892 anglers were recruited to keep an angling diary and participate in the quarterly follow-up calls. Of these participants, 1214 anglers provided information regarding their environmental perceptions and opinions on climate warming.

### Characterization of Participating Anglers

**Fig. 1:**
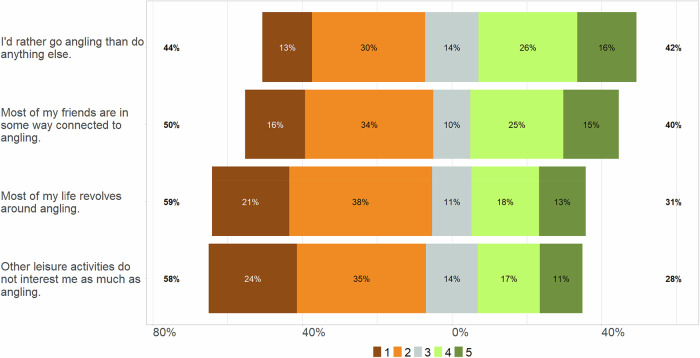
Rating of statements on the centrality-to-lifestyle of angling by the diarists from 1 (strongly disagree), 2 (disagree), 3 (neither), 4 (agree), to 5 (fully agree). The percentages show the proportion of the medium (3) and the lower (1 and 2) and higher (4 and 5) rankings (based on weighted data; unweighted number of participants *n* = 1214)

Most participants were men (91%) and the average age was 48 years (±15 years standard deviation (S.D.)). About 37% of those surveyed had a secondary school leaving certificate and 45% had a high school diploma. Around 58% of the participants were employees, 13% self-employed, and 19% were retired. There were no significant differences between diarists and anglers interviewed in the CATI survey regarding these characteristics (age: MW-U test: *Chi*² = 0.2, *p* = 0.8, education: Wald-test: *F* = 1.2, *p* = 0.3, employment: Wald-test: *F* = 1.2, *p* = 0.3). However, anglers who kept a diary were more avid than those who refused to participate (participants: 28.0 ± 41.2 angling days per year; non-participants: 20.5 ± 35.3 angling days per year; MW-U test: *U* = 7.1, *p* = 0.0001). The majority (59%) of diarists were members of angling clubs, whereas only 49% of the non-participants were angling club members (Wald-test: *F* = 19.3, *p* < 0.0005). Thirty-two percent of the participants fished in flowing waters, 53% in lakes, ponds or channels (standing waters), and 15% in marine waters, primarily the Baltic Sea. The most frequently mentioned target species in the telephone interviews were brown trout (weighted data, 19.9% of participants), pike (18.1%), pikeperch (13.4%), and carp (11.6%) in freshwater and cod (9%) in the Baltic Sea. Pike, pikeperch and carp were mainly fished in standing waters, while trout were mainly fished in rivers and streams. Approximately 37% of the diarists rated their angling skills as average, 44% as above average (median (95% confidence interval) of the skill rating 3 (3, 4), Supplementary Table S1). The overall mean value of the centrality rating was 2.8 (±1.02 S.D.). The number of anglers who disagreed with the statements regarding centrality of angling to their lifestyle exceeded the number of anglers who agreed with the statements (Fig. 1). For example, 59% disagreed that most of their life revolves around angling and 58% that other leisure activities do not interest them as much as angling.

### Motives for Angling

**Fig. 2:**
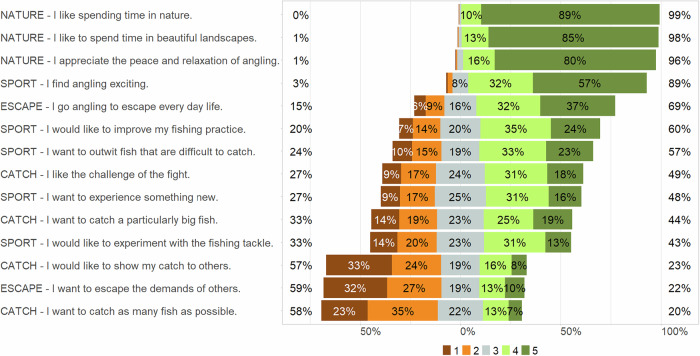
Rating of statements on participants’ motives for angling considered in the EFA from 1 (strongly disagree), 2 (disagree), 3 (neither), 4 (agree), to 5 (fully agree). The percentages show the proportion of the medium (3) and the lower (1 and 2) and higher (4 and 5) rankings (based on weighted data; unweighted number of participants *n* = 1,214). Capital letters indicate the dimensions into which the statements were classified by the EFA

Generally, motives associated with experiencing nature and recreation were rated highest among the participants, while catch-related motives were rated as less important (Fig. 2). The EFA with the best model fit (TLI: 0.91, RMSR: 0.03, RMSEA index: 0.056 (0.049–0.063 90% CI)) explained ~42% of the variance and assigned 14 of the 17 motives to four dimensions: ‘sport’, ‘nature’, ‘catch’, and ‘escape’ (Table 1). The dimension ‘nature’ accounted for most of the variance at 13%. The overall Kaiser–Meyer–Olkin (KMO) factor of 0.78 indicated that a reasonable proportion of the variance could be explained.

**Table 1:** Factor analysis (FA) loadings of the EFA for four underlying dimensions of the motives for angling (sport, nature, catch, escape) as stated by the participants (U: unique variance, H: communality, C: item complexity), along with the medians and 95% confidence intervals (CI) of the ratings of the statements

Statement	Sport (FA 1)	Nature (FA 2)	Catch (FA 3)	Escape (FA 4)	H	U	C	Median (95% CI)
I go angling for sport.	Excluded from the final EFA model							2 (2, 3)
I appreciate the peace and relaxation of angling.	0.04	0.56	–0.07	0.15	0.38	0.62	1.2	5 (5, 5)
I want to spend time with friends or my family.	Excluded from the final EFA model							4 (4, 5)
I want to experience something new.	0.57	–0.03	0.00	0.05	0.3	0.67	1.0	3 (3, 4)
I want to outwit fish that are difficult to catch.	0.42	0.15	0.27	0.00	0.45	0.57	2.0	4 (4, 5)
I like the challenge of the fight.	0.23	0.13	0.40	–0.07	0.33	0.67	2.0	4 (4, 5)
I would like to improve my fishing practice.	0.56	–0.04	0.01	0.04	0.32	0.68	1.0	4 (4, 5)
I go fishing to get fresh fish as food.	Excluded from the final EFA model							3 (3, 4)
I would like to experiment with the fishing tackle.	0.67	–0.04	–0.03	0.05	0.44	0.56	1.0	3 (3, 4)
I like spending time in nature.	-0.01	0.88	–0.01	–0.06	0.75	0.25	1.0	5 (5, 5)
I want to escape the demands of others.	0.03	–0.07	0.12	0.56	0.34	0.66	1.1	2 (2, 3)
I like to spend time in beautiful landscapes.	–0.01	0.73	0.04	0.09	0.56	0.44	1.0	5 (5, 5)
I find angling exciting.	0.42	0.30	0.05	–0.11	0.34	0.66	2.0	5 (5, 5)
I go angling to escape every-day life.	0.05	0.12	–0.06	0.60	0.42	0.58	1.1	4 (4, 5)
I would like to show my catch to others.	0.17	–0.10	0.46	0.00	0.31	0.69	1.4	2 (2, 3)
I want to catch as many fish as possible.	0.02	–0.13	0.53	0.02	0.30	0.70	1.1	2 (2, 3)
I want to catch a particularly big fish.	–0.05	0.03	0.79	0.02	0.60	0.40	1.0	3 (3, 4)

### Rating of NEP Statements

**Fig. 3:**
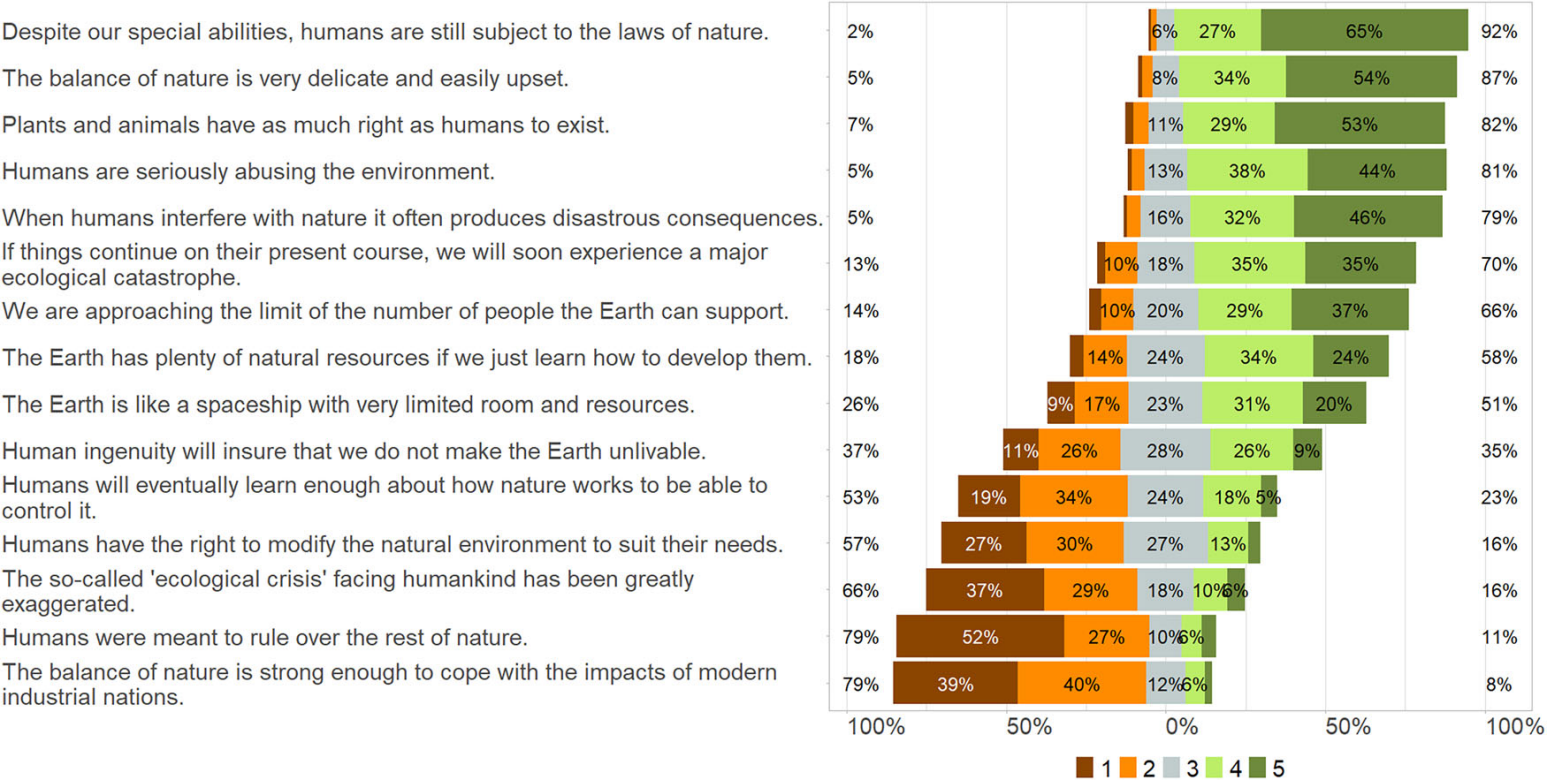
Rating of statements on the New Environmental Paradigm (NEP) scale by participants from 1 (strongly disagree), 2 (disagree), 3 (neither), 4 (agree), to 5 (fully agree). The percentages show the proportion of the medium (3) and the lower (1 and 2) and higher (4 and 5) rankings (based on weighted data; unweighted number of participants *n* = 1214)

The NEP statements cover five facets of environmental attitudes: balance of nature, the possibility of an eco-crisis occurring, antiexemtionalism (belief that humans are not exempt from the constraints of nature), limits to growth (belief that the resources of the earth are limited), and anti-anthropocentrism (belief that humans have not the right to control the natural environment) (Amburgey and Thoman, 2012). The Cronbach alpha of 0.71 (0.69, 0.73 C.I.) indicated good internal consistency of the 15 statements, which confirmed that the NEP scale measures a single concept and can be treated as a single variable in the regression analyses. The analysis revealed a wide variety of environmental attitudes among the participating anglers. Nonetheless, NEP statements associated with anthropocentrism and doubts about the eco-crisis received relatively low approval ratings (Supplementary Table S2). For example, only 11% of the participants agreed that humans were meant to rule over the rest of nature (median and 95% confidence interval (CI) of all ratings: 1 (1, 2)), and only 8% assumed that the balance of nature would be strong enough to cope with the impacts of modern industrial nations (median and 95% CI of all ratings: 2 (2, 3)). In contrast, statements regarding nature’s sensitivity and the need to protect it received a comparably high level of approval (Supplementary Table S2). For instance, around 87% of participants agreed that the balance of nature is very fragile, delicate, and easily disturbed (median and 95% CI of all ratings: 5 (5,5)) (Supplementary Table S2), and around 70% predicted that a major ecological catastrophe would occur if things would continue on their present course. Statements opposing exemptionalism also received a high level of approval (Fig. 3). For example, 92% of participants agreed that, despite their special abilities, humans are still subject to the laws of nature (median and 95% CI of all ratings 5 (5,5)), whereas around one third of participants were optimistic about human ingenuity and agreed that human ingenuity would prevent the Earth from becoming uninhabitable (median and 95% CI of all ratings: 3 (3, 4)). Accordingly, 58% of participants agreed that the Earth has plenty of natural resources if we just learn how to develop them, whereas 51% agreed that room and resources of the Earth are very limited.

### Views on Climate Warming

The majority of participants (61.4%, *n* = 709.3, weighted data) agreed that climate warming is already affecting angling in Germany. Twenty-nine percent (*n* = 336.9, weighted data) disagreed, 0.08% (*n* = 1.4, weighted data) denied the existence of climate change, and 9% (*n* = 107.4, weighted data) stated that they could not make a judgement. The likelihood of recognising the impact of climate change on angling increased particularly among anglers with a pro-environmental attitude (mean NEP ratings), and increased slightly among those for whom sport-related motives were important (Table 2).

**Table 2:** Results of the final binomial regression analysis regarding the first statement: “Are climate changes already having an impact on angling in Germany?”. Reference category: “No impact”. Anglers who denied climate warming or answered with “neither” were not considered in the analysis

Term	Estimate	S.E.	Statistic	Odds ratio	*p*
Intercept	–0.83	0.93	–0.89	0.44	0.4
Age	–0.02	0.006	–2.62	0.98	0.009
Skill	0.16	0.09	1.89	1.18	0.06
Gender male	–0.93	0.51	–1.85	0.39	0.07
Motive sport	0.26	0.09	2.79	1.3	0.005
NEP (mean)	0.73	0.19	3.88	2.07	0.0001
Nagelkerke *r*²		0.09			
Hosmer Lemeshow test		*Chi*² = 7.5	df = 8	*p* = 0.5	
Likelihood ration test		*Chi*² = 42.9	df = 5	*p* < 0.0001	
Accuracy		69%			

**Fig. 4:**
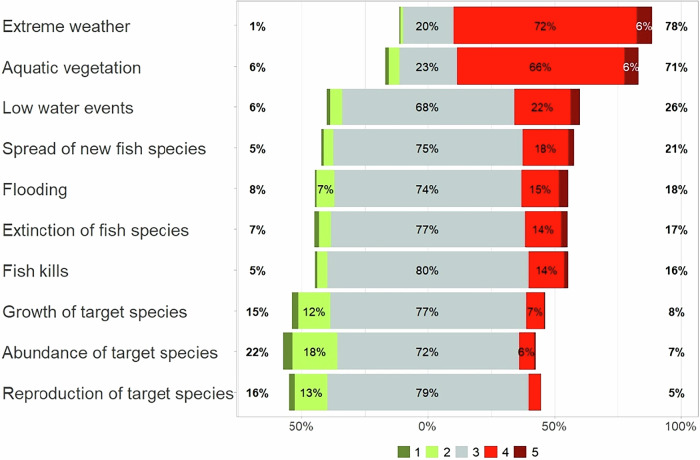
Participants’ assessment of the already observed impacts of climate warming on weather events and aquatic ecosystems from 1 (has strongly decreased), 2 (has decreased), 3 (neither), 4 (has increased), to 5 (has strongly increased)

**Fig. 5:**
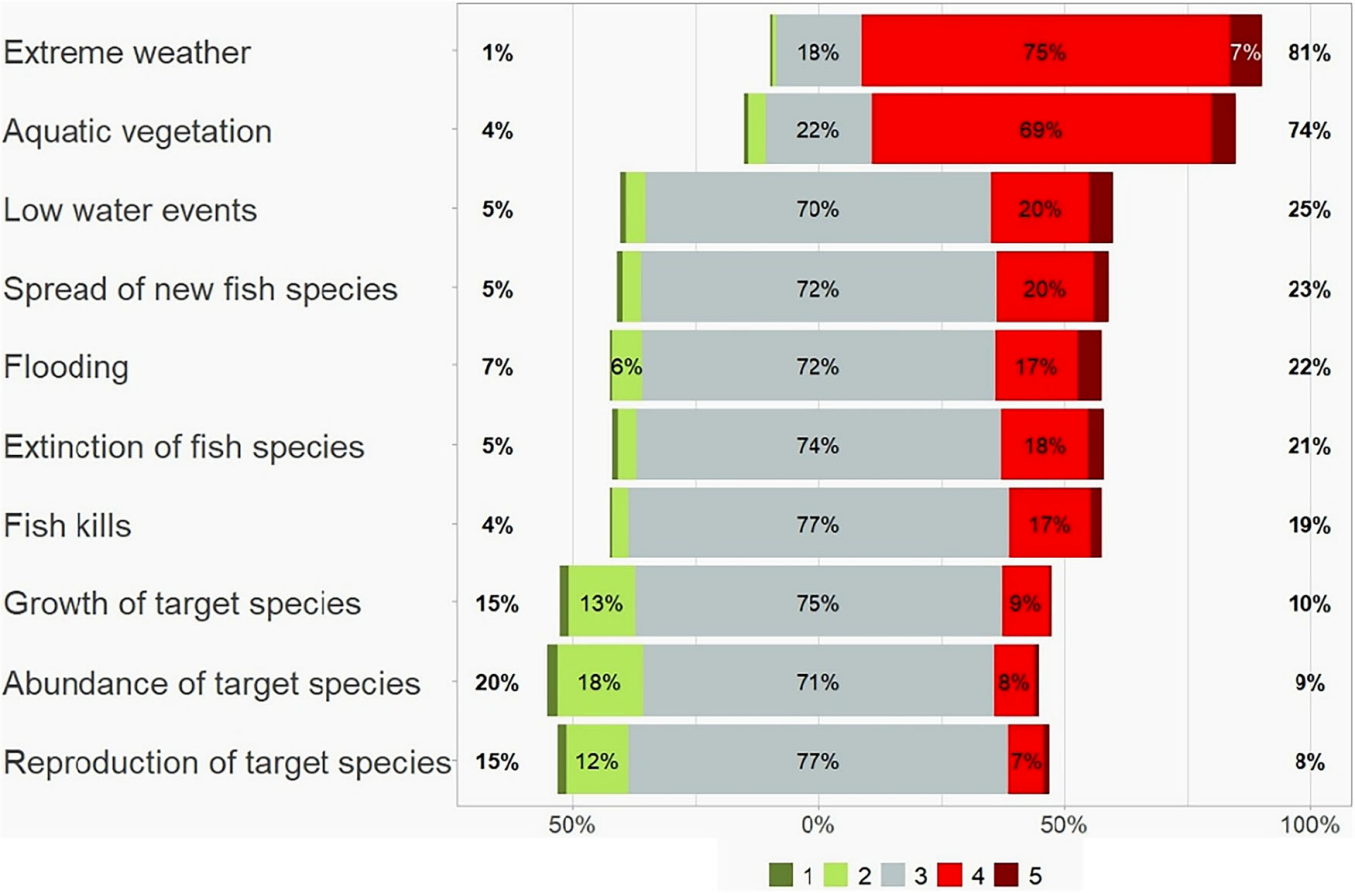
Participants’ assumptions of the future impacts of climate warming on weather events and aquatic ecosystems from 1 (will strongly decrease), 2 (will decrease), 3 (neither), 4 (will increase), to 5 (will strongly increase)

Most anglers assumed that climate warming would primarily affect the weather and aquatic vegetation, with comparativley less impact on fish species or water levels. A majority did not observe effects of climate change on water levels, the spread of new fish species, fish kills, the extinction of fish species, or the growth, abundance, and reproduction of target species. About 80% and 70% of participating anglers, respectively, stated that extreme weather and phytoplankton blooms/aquatic vegetation have increased due to climate warming (Fig. 4). In addition, a majority of the participants suspected that climate warming might lead to further increases of extreme weather events and aquatic vegetation growth in the future. In contrast, only a minority of anglers observed or predicted the impact of climate warming on low water levels, flooding events, and fish species (Figs. 4, and 5). Around 16% of anglers suspected that fish kills had become more frequent, and 19% assumed that they will become more frequent in the future. Furthermore, 17% suspected an increased extinction of fish species, while 21% assumed that the extinction of fish species will become more frequent in the future. An increased spread of new fish species was assumed by 21% of participants, and 23% believed this would occur in the future. Negative impacts of climate warming on growth, abundance, and reproduction of their target species were observed by 15%, 22%, and 16% of anglers, respectively. A similar proportion assumed that these impacts would continue in the future. Approximately 10% of participants believed that climate warming will have a positive impact on the growth of their target species. Accordingly, a majority of participants considered it unlikely or very unlikely that their angling behaviour would change as a result of climate warming. However, 65% stated that they would likely or very likely adapt to possible changes in the angling season. Less than 5% assumed that they would likely or very likely stop angling due to climate changes (see Supplementary Table S1 for details of the ratings).

Overall, the ordinal regression analyses indicated that age, education level, gender, membership in angling clubs, avidity, centrality, preferred angling water, and motives for angling had only a small influence on the recognition of global-warming-related effects on aquatic ecosystems. Systematic patterns, however, were rarely observed. Anglers with high NEP scores were more likely to assume that changes in aquatic ecosystems had already occurred and/or to expect such changes in the future. As NEP scores increased, so did the likelihood of reporting increases in global-warming-related effects on the loss of native species, growth of target species, fish kills, phytoplankton blooms, low-water events, and extreme weather conditions. Higher NEP scores were also associated with a greater likelihood of assuming future increases in the spread of new species, loss of native fish species, and the frequency of fish kills, phytoplankton blooms, low water events, and extreme weather conditions. Although model fit tests indicated that the analysis yielded reasonable fits, the generally low Nagelkerke *r*² values between 0.03 and 0.09 indicated weak relationships between predictors and outcome variables. The high frequency of the response ‘neither’ in eight of the ten questions may also have contributed to the low explanatory power of the models, and it cannot be ruled out that the large sample size (*n* = 1163) partially influenced the *p*-values. The results and model fits of the regression analyses are presented in detail in the supplementary material (Supplementary Table S4; Table S5).

The preferred angling waters had little influence on the assessment of climate warming impacts. The results and model fits of the regression analyses are presented in detail in the supplementary material (Supplementary Tables S4;and S5). When participants were grouped according to their favorite target species (carp, pike, pikeperch, cod and salmonid), only minor significant differences emerged between the groups regarding five statements on the effects of climate change. Compared to other groups, carp anglers were less likely to agree that the abundance of their target species had decreased or would decrease in the future. Cod, pikeperch, and salmonid anglers did not report an increase in the growth of their target species due to climate change. Moreover, compared to pike and salmonid anglers, carp and pikeperch anglers were less likely to assume that they would stop angling due to climate change (see supplementary material Supplementary Figs. S1–S5).

## Discussion

### Anglers’ Assessments of Climate Warming Impacts on Aquatic Ecosystems

Pro-environmental attitudes are predictors of pro-environmental behaviour (Miller et al., 2022), although contextual factors, including societal, political, socio-cultural, and cognitive or physical constraints, can influence the translation of these attitudes into corresponding behaviour (Steg and Vleg, 2009; Wyss et al., 2022). The NEP scores indicated that most participating anglers were aware of the sensitivity of nature, regardless of differences in socio-demographic characteristics or motives for angling. Accordingly, the overwhelming majority of anglers acknowledged the existence of global warming, and most participants suspected a link between global warming and the frequency of extreme weather events. This finding may also have been influenced by the summer storms and extreme weather events that struck western Europe during the study period in 2021, as experience of environmental changes can reinforce beliefs in climate warming (Reser and Bradley, 2020; Damsbo-Svendsen, 2021). When asked about specific effects of global warming on aquatic ecosystems, most participants attributed increased macrophyte growth and phytoplankton blooms to climate warming and assumed that such events would become more frequent in the future. These assessments are consistent with scientific studies confirming the impact of global warming on phytoplankton development (Winder and Sommer, 2012; Paerl and Paul, 2012) and macrophyte growth (Silveira and Thiébaut, 2017).

In contrast, only a minority of participants recognised impacts of global warming on water levels or expected such impacts to occur in the future, which suggests a lack of experience or knowledge about the relationships between weather events and runoff dynamics. However, the ways in which people experience climate-warming-related events and their general risk perceptions interact in complex ways. These perceptions are influenced by various factors, including prior beliefs, personal experiences, political orientations, and social norms (Echavarren et al., 2019; Brügger et al., 2021; Howe, 2021).

Surprisingly, most participants had not observed any effects of global warming on the abundance, growth or reproduction of their target fish species, nor did they expect to see any such effects in the future, despite various studies attributing profound changes in fish communities, including shifts in body size, age structure and species composition, to global warming in Germany and across Europe (Simpson et al., 2011; Jeppesen et al., 2012; Le Hen et al., 2023). In contrast to the anglers in this study (Supplementary Table S4), around 60% of Australian marine anglers recognised changes in the species composition and distributional ranges of the fish they caught (Ryan et al., 2022).

The participating anglers documented an average of 11 fishing trips (±14 S.D.) in their diaries. Furthermore, 57% of participants had started fishing before the age of 14, suggesting that many of them were experienced anglers. Nonetheless, gradual or subtle ecological shifts might be difficult to detect and track based on personal experience (Moser, 2010), particularly in the presence of large temporal and/or geographic distances between causes and effects (Weber, 2010). Moreover, anglers may overlook climate-related environmental information (McDonald et al., 2015) or attribute their observations to other causes, partly because the dynamics of complex ecosystems are characterised by multiple feedback loops, time delays, and non-linearities (Hewitt et al., 2015; Jaiswal et al., 2021). Although climate warming has global impacts, observed and predicted impacts can vary widely from region to region, as the direction and magnitude of these impacts depend on whether warming moves local temperatures into, beyond, or away from the optimal ranges (Eltahir and Choi, 2025). Additionally, because anglers target only a limited number of species, often large predators (Flink et al., 2024), they may not immediately notice changes in the broader fish community. Anglers who target fish species such as carp, which may even benefit from climate warming (Souza et al., 2022), may consider warming effects as less relevant or may not have observed any negative changes. Furthermore, the subjective attribution of observed changes in aquatic ecosystems to global warming is significantly predicted by pre-existing beliefs about climate change, political attitudes, and perceived normative cues (Whitman et al., 2018; Ogunbode et al., 2019). As noted above, the vast majority of the participants of this study were aware of climate warming. However, Spence et al. (2012) conducted a representative study on public perceptions of climate change in the UK and showed that, although the majority were aware of climate change, many participants were unsure of its impacts and considered it possible that these impacts had been exaggerated. The most probable causes for these perceptions were psychological distance, media reporting and uncertainties in scientific analyses. Finally, although it is a controversial topic (van Valkengoed et al., 2023), people in industrialised countries may psychologically distance themselves from the impacts of climate warming, which prevents them from being fully aware of climate change-related issues (McDonald et al., 2015). This detachment may arise from the perception that climate change affects distant places and future generations more severely, leading to the judgement that personal risks are lower than societal risks (Spence et al., 2012). The fact that relatively few respondents anticipated an impact of global warming on fish assemblages, either now or in the future, may explain why most anglers believed they could adapt their hobby to climate-related changes. Only a minority thought they would have to stop angling altogether (Supplementary Table S3).

### Factors Influencing the Assessments of Climate Related Impacts

The gender distribution and age of the participating anglers were similar to those documented in other surveys of anglers (Thunberg and Fulcher, 2006; Gundelund et al. 2020). The level of education was commensurate with the German population average, with more than 80% of 25- to 64-year-olds holding at least an upper secondary school qualification (OECD, 2014).

Gender, education and age are known to influence environmental awareness and risk perception (Meyer, 2015; Poortinga et al., 2019). However, unlike a cross-European representative study which found positive associations between educational attainment and concern about climate warming and negative associations between age and concern (Poortinga et al., 2019), this study found no substantial effect of age or education level on anglers’ views on climate warming. The impact of age on climate concern, however, has been assessed with mixed results in previous research. A recent online survey conducted in Israel indicated that older people are significantly more concerned about climate change and more likely to acknowledge the existence of climate warming than younger people (Ayalon, 2024). An earlier meta-analysis indicated that the influence of age on environmental concern was generally positive but negligible, which was attributed to stronger influence of social norms and traditional values (Wiernik et al., 2013). Other researchers also pointed out that environmental values and political orientation were better predictors for environmental concern than age, whether defined as individual age or generational cohort (Goto Gray et al., 2019). This study also found no clear evidence that gender influences observed or assumed impacts of climate warming. Although there are indications that, for example, differences in socialisation and gender-class effects may foster a deeper concern for the environment among women (Eisler et al., 2003; Goldsmith et al., 2013; Poortinga et al., 2019; Echavarren, 2023), study results regarding the influence of gender on environmental perceptions are inconclusive (Henshall Momsen, 2000), most likely because the influence of gender is shaped by broader societal, political, socio-cultural, and economic factors (Pearson et al., 2017; Economou and Halkos, 2020; Ergun et al., 2024).

The results of this study confirm other studies indicating that people engage in angling for multiple reasons including angling-related (nature experience, escape from everyday life) and angling-specific motives (catching fish), with some anglers valuing nature experience higher than angling-specific motives (Fedler and Ditton, 1994; Gundelund et al., 2022). Contrary to our expectations, however, angling motives as well as angling club membership rarely influenced the views on global warming. Other studies have indicated positive correlations between nature experience as a motivation to fish, membership of angling clubs, and a stronger sense of responsibility towards conservation issues (Copeland et al., 2017; van den Heuvel et al., 2020). Identification with an activity can encourage pro-environmental behaviour and the internalisation of moral and normative beliefs about acting as stewards of aquatic resources (Landon et al., 2018). The specialisation of anglers, as measured by skill, avidity and anglers’ centrality (Koemle et al., 2024), fell within the ranges observed in other studies of German anglers (Arlinghaus and Mehner, 2004; Lewin et al., 2021). Specialisation rarely influenced the ratings of the statements regarding climate warming. As hypothesised, anglers with higher NEP scores were more likely to attribute observed or anticipated changes to global warming, consistent with research showing that individuals with pro-environmental attitudes tend to show greater interest in environmental issues and express more concern about global warming (Poortinga et al., 2004; Steel et al., 2005; Halkos and Matsiori, 2017). However, the low explanatory power of the regression models suggests that anglers’ recognition of global warming is influenced by additional factors not considered in this study. The relationship between environmental attitudes, ecological knowledge and perception of environmental risks is shaped by, for example, cultural worldview dimensions, and higher scores on egalitarianism and lower scores on hierarchism and individualism are associated with higher levels of perceived environmental risk (Stevenson et al., 2014; Johansson et al., 2022). Including these predictors along with variables such as place attachment, cultural beliefs, urban or rural residency, norms and values, political attitude, and social class may lead to more comprehensive insights (Weber, 2016; Ballew et al., 2020; Brick and Lai, 2018).

### Study Limitations

Some shortcomings are important to consider when interpreting the results of this study. The anglers who participated in this study were recruited from the general population in Germany through a representative telephone survey. However, anglers who choose to participate in scientific studies may differ from those who refuse to participate (Connelly and Brown 1995). Despite the small socio-demographic differences between the ‘general’ angler population, as identified by the initial telephone screening, and the participants of this study, there may be differences in pro-environmental attitudes between the two groups. In addition, self-reported measures in surveys on environmental attitudes may be subject to social desirability bias (Milfont, 2009). Even though empirical evidence suggests that the impact of social desirability on environmental research is relatively small, this possible bias should be considered (Milfont, 2009). Moreover, the NEP scale can be used to uncover factors underlying the participants’ environmental attitudes (Dunlap et al., 2000; Amburgey and Thoman, 2012; Halkos and Matsiori, 2017). Furthermore, since anglers were asked to what extent climate warming has already affected fish stocks, it cannot be ruled out that some participants observed changes in fish stocks but did not attribute them to climate warming. Finally, generalizing the results to anglers in other countries may be challenging because the relationship between climate change perceptions and predictor variables can vary across nations (Poortinga et al., 2019).

### Conclusions and Future Research Needs

Specialised anglers often voluntarily act as stewards of their resources (Granek et al., 2008; Oh and Ditton, 2008) and engage in environmental monitoring programmes that can complement scientific monitoring (Williams et al., 2016; Tsuboi et al., 2024). Moreover, anglers’ local ecological knowledge can serve as an early warning system for detecting rapid or unforeseen changes in highly dynamic ecosystems, enabling early action (Lewin et al., 2025). The millions of anglers worldwide thus represent an enormous, largely untapped potential for monitoring the impacts of climate warming. The results of this study indicated that, although anglers should not be regarded as a homogenous group, most socio-demographic distinctions can be ignored when recruiting participants for citizen science projects aimed at detecting signs of climate warming. However, as with other projects of this kind, specific training programmes should be developed to improve relevant ecological skills and ensure standardised monitoring methods. One example is the ‘Flow Project’ (https://www.flow-projekt.de/index.php/de/), a Germany-wide citizen science project which uses video tutorials, identification booklets, and online quizzes to train citizens to assess the ecological status of small streams by collecting data on macroinvertebrates and stream hydro-morphology (Von Gönner et al., 2023, 2024). The training of anglers could focus on indicators of climate warming with regard to the fish communities. Anglers are particularly well suited to monitor the fish fauna as this activity requires specialised equipment and a fishing licence. Relevant indicators might include the decline or loss of cold- and cool-water species such as salmonids, increased populations or self-reproducing eurytherm carp, the occurrence of non-native fish species, and changes in length or age structure of target fish species. Citizen science data can contribute meaningfully to studies on the effects of climate change when compared with data from other sources (see McDonald et al. 2025 as example). Furthermore, participation in citizen science projects can encourage individual and collective pro-environment behavior, motivating participants to engage in place-based measures that support local ecosystem functioning and reduce the effects of climate change—for example, through habitat restoration, the protection or creation of diverse habitat types, or changes in angling practices to help protect targeted species (Palmer et al., 2009; Jeanson et al., 2021). Angling clubs can serve as valuable partners in this process (Brownscombe et al., 2019). However, further research is needed to develop methods that ensure quality assurance, validation and standardisation of data collection and documentation by the citizen scientists according to scientific standards (Delaney et al., 2008; Aceves-Bueno et al., 2015).

## Supplementary information

Supplementary information

## Data Availability

Personalized raw data for are not publicly available to preserve individuals’ privacy under the European General Data Protection Regulation. Anonymized data will be made available on request.
