# Supplementary material for: German Anglers’ Views on Global Warming – Implications for Climate Change Monitoring and Management
Source: Environ Manage. 2025 Dec 1;76(1):20. doi: 10.1007/s00267-025-02291-2 (PMC12669331; doi:10.1007/s00267-025-02291-2)
Supplement: Supplementary file 1 — Supplementary information [file 267_2025_2291_MOESM1_ESM.docx]

**Supplementary material**

*Detailed information on the weighting procedure*

Prior to the analyses, the data were weighted to enable generalization of the conclusions drawn from the analyses. Since the telephone sample for the household screening was drawn using a dual-frame mode with a mixture of landline and mobile phone numbers, design weighting for the two subsamples, "mobile" and "landline", was carried out first. As households were contacted first in the survey, redressment weighting was then applied to correct the accessibility dependent on household size. An additional weighting procedure was used to account for disproportional sampling across the 16 German federal states. Finally, the household samples were converted into personal samples. As certain groups, such as highly committed or educated people, are more likely to participate in such surveys (Taylor and Ryan, 2020), adjustment weighting was performed to align the participants with the average angler population for the diary data. This was done using data from the initial household screening survey. The weighting was carried out using the variables age, education, avidity, proximity to the coast, and membership in angling clubs, that had been significant in a previously conducted generalized linear model (GLM with "logit" link) to explain factors influencing full participation in the diary study.

Suppl. Tab. S1: Rating of statements (median and 95% confidence intervals (CI)) regarding skill level (1: beginner, 2: slightly below average, 3: average, 4: slightly above average, 5: expert) and centrality (1: strongly disagree, 2: disagree, 3: neither, 4: agree, 5: fully agree).

| Statement | Median | 95% CI |
| --- | --- | --- |
| Skill level |  |  |
| How would you rate your fishing skills compared to the average angler? | 3 | 3, 4 |
| I would describe myself as a fishing expert. | 2 | 2, 3 |
| Centrality |  |  |
| A large part of my life revolves around fishing. | 2 | 2, 3 |
| I'd rather go fishing than do anything else. | 2 | 2, 3 |
| Other hobbies don't interest me as much as fishing. | 2 | 2, 3 |
| Most of my friends are also involved in fishing. | 2 | 2, 3 |

Suppl. Tab. S2: Rating of statements (median and 95% confidence intervals (CI)) of the New Environmental Paradigm (NEP) scale (1: strongly disagree, 2: disagree, 3: neither, 4: agree, 5: fully agree).

| Statement | Median (95% CI) | Dimension  (Amburgey and Thoman, 2012) |
| --- | --- | --- |
| We are approaching the limit of the number of people the Earth can support. | 4 (4, 5) | Limits to growth |
| Humans have the right to modify the natural environment to suit their needs. | 2 (2, 3) | Antianthropocentism |
| When humans interfere with nature it often produces disastrous consequences. | 4 (4, 5) | Balance of nature |
| Human ingenuity will ensure that we do not make the Earth unliveable. | 3 (3, 4) | Antiexemptionalism |
| Humans are seriously abusing the environment. | 4 (4, 5) | Ecocrisis |
| The Earth has plenty of natural resources if we just learn how to develop them. | 4 (4, 5) | Limits to growth |
| Plants and animals have as much right as humans to exist. | 5 (5, 5) | Antianthropocentism |
| The balance of nature is strong enough to cope with the impacts of modern industrial nations. | 2 (2, 3) | Balance of nature |
| Despite our special abilities, humans are still subject to the laws of nature. | 5 (5, 5) | Antiexemptionalism |
| The so-called “ecological crisis” facing humankind has been greatly exaggerated. | 2 (2, 3) | Ecocrisis |
| The Earth is like a spaceship with very limited room and resources. | 4 (3, 4) | Limits to growth |
| Humans were meant to rule over the rest of nature. | 1 (1, 2) | Antianthropocentism |
| The balance of nature is very delicate and easily upset. | 5 (5, 5) | Balance of nature |
| Humans will eventually learn enough about how nature works to be able to control it. | 2 (2, 3) | Antiexemptionalism |
| If things continue on their present course, we will soon experience a major ecological catastrophe. | 4 (4, 5) | Ecocrisis |

Amburgey, J.W., Thoman, D.B., 2012. Dimensionality of the New Ecological Paradigm: Issues of factor structure and measurement. Environment and Behavior 44, 235-256. https://doi.org/10.1177/0013916511402.

Suppl. Tab. S3: Rating of statements (median and 95% confidence intervals (CI)) regarding observed and predicted impacts of climate change on aquatic ecosystems, target species, weather conditions, and future angling activities (1: has strongly decreased/will strongly decrease, 2: has decreased/will decrease, 3: neither, 4: has increased/will increase, 5: has strongly increased/will strongly increase; *: 1: very unlikely, 2: unlikely, 3: neither, 4: likely, 5: very likely; based on weighted data).

| Statement | Median | 95% CI |
| --- | --- | --- |
| How have climate changes in recent years influenced the spread of new fish species? | 3 | 3, 4 |
| How will climate changes influence the spread of new fish species? | 3 | 3, 4 |
| How have climate changes in recent years already influenced the extinction of fish species? | 3 | 3, 4 |
| How will climate changes influence the extinction of fish species? | 3 | 3, 4 |
| To what extent have climate changes in recent years already influenced the reproduction of your target fish species? | 3 | 3, 4 |
| To what extent will climate changes influence the reproduction of your target fish species? | 3 | 3, 4 |
| To what extent have climate changes in recent years already influenced the abundance of your target fish species? | 3 | 3, 4 |
| To what extent will climate changes influence the abundance of your target fish species? | 3 | 3, 4 |
| To what extent have climate changes in recent years already influenced the growth of your target fish species? | 3 | 3, 4 |
| To what extent will climate changes influence the growth of your target fish species? | 3 | 3, 4 |
| To what extent have climate changes in recent years already led to fish kills? | 3 | 3, 4 |
| To what extent will climate changes lead to fish kills? | 3 | 3, 4 |
| To what extent have climate changes in recent years already influenced aquatic vegetation? | 4 | 4, 5 |
| To what extent will climate changes influence aquatic vegetation? | 4 | 4, 5 |
| To what extent have climate changes in recent years already led to low water events? | 3 | 3, 4 |
| To what extent will climate changes lead to low water events? | 3 | 3, 4 |
| To what extent have climate changes in recent years already led to flooding? | 3 | 3, 4 |
| To what extent have climate changes in recent years already led to flooding? | 3 | 3, 4 |
| To what extent have climate changes in recent years already led to extreme weather? | 4 | 4, 5 |
| To what extent will climate changes lead to extreme weather? | 4 | 4, 5 |
| I will be angling more often due to climatic changes. | 2 | 2, 3 |
| I will be angling less often due to climatic changes. | 2 | 2, 3 |
| I will spend more money on angling due to climatic changes. | 2 | 2, 3 |
| I will adapt to changes in the fishing season due to climatic changes. | 4 | 4, 5 |
| I will change my target fish species due to climatic changes. | 2 | 2, 3 |
| I will change my angling waters due to climatic changes. | 2 | 2, 3 |
| I will change my angling method due to climatic changes. | 2 | 2, 3 |
| I will stop angling due to climatic changes | 1 | 1, 2 |

Suppl. Tab. S4: Results of final ordinal regression models applied to investigate angler related factors that might have influenced the statements regarding observed or assumed impacts of climate warming on angling and the aquatic ecosystems (S.E.: standard error, OR: odds ratio, Thr. coeff: threshold coefficient, Edu2: secondary school, Edu3: high school diploma, reference category: Edu1 (primary school), Angl. day: number of angling days 12 month preceding the survey, Angl. club: member of angling club, reference category: no membership).

| To what extent have climate changes in recent years already influenced the following aspects of your main fishing water: | | | | | | To what extent will climate changes influence the following aspects of your main fishing water: | | | | | |
| --- | --- | --- | --- | --- | --- | --- | --- | --- | --- | --- | --- |
| Spread of new fish species | Estimate | S.E. | z | OR | p | Spread of new fish species | Estimate | S.E. | z | OR | p |
| Age | - | - | - | - | - | Age | -0.01 | 0.005 | -1.85 | 0.99 | 0.07 |
| Edu2 | 0.04 | 0.24 | 0.15 | 1.04 | 0.9 | Edu2 | -0.2 | 0.23 | -0.85 | 0.82 | 0.39 |
| Edu3 | 0.4 | 0.23 | 1.7 | 1.49 | 0.08 | Edu3 | 0.12 | 0.23 | 0.52 | 1.13 | 0.6 |
| Gender male | - | - | - | - | -- | Gender male | - | - | - | - | - |
| Angl. day | - | - | - | - | - | Angl. day | - | - | - | - | - |
| Angl. club | 0.24 | 0.17 | 1.47 | 1.28 | 0.1 | Angl. club | 0.42 | 0.16 | 2.67 | 1.52 | 0.008 |
| Centrality | 0.24 | 0.09 | 2.81 | 1.27 | 0.005 | Centrality | 0.21 | 0.08 | 2.65 | 1.24 | 0.008 |
| Marine water | -0.5 | 0.23 | -2.18 | 0.6 | 0.03 | Marine water | - | - | - | - | - |
| Standing water | -0.3 | 0.16 | -1.92 | 0.73 | 0.06 | Standing water | - | - | - | - | - |
| Motive catch | - | - | - | - | - | Motive catch | - | - | - | - | - |
| Motive sport | 0.16 | 0.08 | 1.98 | 1.18 | 0.05 | Motive sport | - | - | - | - | - |
| Motive nature | - | - | - | - | - | Motive nature | - | - | - | - | - |
| Motive escape | - | - | - | - | - | Motive escape | - | - | - | - | - |
| NEP | 0.26 | 0.16 | 1.65 | 1.3 | 0.09 | NEP | 0.36 | 0.16 | 2.31 | 1.44 | 0.02 |
| SKILL | - | - | - | - | - | SKILL | - | - | - | - | - |
| Thr. coeff. 1-2 | -2.23 | 0.73 | -3.04 | 0.11 |  | Thr. coeff. 1-2 | -2.51 | 0.76 | -3.3 | 0.08 | - |
| Thr. coeff. 2-3 | -0.76 | 0.7 | -1.1 | 0.47 |  | Thr. coeff. 2-3 | -0.96 | 0.71 | -1.34 | 0.38 | - |
| Thr. coeff. 3-4 | 2.28 | 0.7 | 3.27 | 9.8 |  | Thr. coeff. 3-4 | 2.05 | 0.71 | 2.87 | 7.74 | - |
| Thr. coeff. 4-5 | 5.05 | 0.73 | 6.96 | 156.7 |  | Thr. coeff. 4-5 | 4.78 | 0.74 | 6.5 | 119.6 | - |
| Loss of fish species |  |  |  |  |  | Loss of fish species |  |  |  |  |  |
| Age | -0.01 | 0.005 | -2.93 | 0.98 | 0.003 | Age | -0.02 | 0.005 | -3.98 | 0.98 | < 0.0001 |
| Edu2 | -0.46 | 0.24 | -1.92 | 0.63 | 0.06 | Edu2 | -0.33 | 0.24 | -1.4 | 0.72 | 0.2 |
| Edu3 | 0.003 | 0.24 | 0.01 | 1.0 | 0.9 | Edu3 | 0.1 | 0.23 | 0.43 | 1.11 | 0.7 |
| Gender male | -0.69 | 0.38 | -1.82 | 0.5 | 0.07 | Gender male | -0.91 | 0.39 | -2.35 | 0.4 | 0.02 |
| Angl. day | - | - | - | - | - | Angl. day | - | - | - | - | - |
| Angl. club | -0.22 | 0.16 | -1.41 | 0.8 | 0.2 | Angl. club | - | - | - | - | - |
| Centrality | - | - | - | - | - | Centrality | - | - | - | - | - |
| Marine water | - | - | - | - | - | Marine water | 0.32 | 0.22 | 1.42 | 1.38 | 0.2 |
| Standing water | - | - | - | - | - | Standing water | -0.11 | 0.16 | -0.69 | 0.89 | 0.5 |
| Motive catch | - | - | - | - | - | Motive catch | - | - | - | - | - |
| Motive sport | - | - | - | - | - | Motive sport | - | - | - | - | - |
| Motive nature | - | - | - | - | - | Motive nature | - | - | - | - | - |
| Motive escape |  |  | - | - | - | Motive escape | - | - | - | - | - |
| NEP | 0.36 | 0.16 | 2.23 | 1.43 | 0.03 | NEP | 0.43 | 0.16 | 2.74 | 1.55 | 0.006 |
| SKLL | 0.16 | 0.07 | 2,28 | 1.18 | 0.02 | SKLL | - | - | - | - | - |
| Thr. coeff. 1-2 | -3.36 | 0.83 | -4.06 | 0.04 |  | Thr. coeff. 1-2 | -4.45 | 0.82 | -5.37 | 0.01 |  |
| Thr. coeff. 2-3 | -2.01 | 0.81 | -2.49 | 0.13 |  | Thr. coeff. 2-3 | -2.83 | 0.79 | -3.58 | 0.06 |  |
| Thr. coeff. 3-4 | 1.08 | 0.8 | 1.35 | 2.95 |  | Thr. coeff. 3-4 | 0.23 | 0.78 | 0.29 | 1.23 |  |
| Thr. coeff. 4-5 | 3.43 | 0.82 | 4.17 | 30.75 |  | Thr. coeff. 4-5 | 2.62 | 0.79 | 3.3 | 13.77 |  |
| Reproduction of target species | |  |  |  |  | Reproduction of target species | |  |  |  |  |
| Age | -0.02 | 0.005 | -3.97 | 0.98 | < 0.0001 | Age | -0.007 | 0.005 | -1.38 | 0.99 | 0.2 |
| Edu2 | - | - | - | - | - | Edu 2 | - | - | - | - | - |
| Edu3 | - | - | - | - | - | Edu 3 | - | - | - | - | - |
| Gender male | 1.0 | 0.37 | 2.69 | 2.71 | 0.007 | Gender male | 1.24 | 0.37 | 3.41 | 3.46 | 0.0006 |
| Angl. day | - | - | - | - | - | Angl. Day | - | - | - | - | - |
| Angl. club | - | - | - | - | - | Angl. Club | - | - | - | - | - |
| Centrality | - | - | - | - | - | Centrality | - | - | - | - | - |
| Marine water | 0.58 | 0.24 | 2.45 | 1.78 | 0.01 | Marine water | 0.17 | 0.23 | 0.74 | 1.19 | 0.5 |
| Standing water | 0.58 | 0.17 | 3.45 | 1-78 | 0.006 | Standing water | 0.23 | 0.17 | 1.35 | 1.25 | 0.2 |
| Motive catch | - | - | - | - | - | Motive catch | - | - | - | - | - |
| Motive sport | - | - | - | - | - | Motive sport | - | - | - | - | - |
| Motive nature | - | - | - | - | - | Motive nature | 0.14 | 0.07 | 1.98 | 1.15 | 0.047 |
| Motive escape | - | - | - | - | - | Motive escape | - | - | - | - | - |
| NEP | - | - | - | - | - | NEP | -0.13 | 0.16 | -0.78 | 0.88 | 0.4 |
| SKILL | - | - | - | - | - | SKILL | - | - | - | - | - |
| Thr. coeff. 1-2 | -2.93 | 0.49 | -5.97 | 0.05 |  | Thr. coeff. 1-2 | -2.78 | 0.78 | -3.58 | 0.06 |  |
| Thr. coeff. 2-3 | -0.67 | 0.47 | -1.43 | 0.51 |  | Thr. coeff. 2-3 | -0.6 | 0.77 | -0.79 | 0.55 |  |
| Thr. coeff. 3-4 | 2.61 | 0.48 | 5.47 | 13.61 |  | Thr. coeff. 3-4 | 2.33 | 0.77 | 3.04 | 10.32 |  |
| Thr. coeff. 4-5 | 5.15 | 0.62 | 8.37 | 172.6 |  | Thr. coeff. 4-5 | 4.8 | 0.82 | 5.84 | 122.5 |  |
| Abundance of target species | |  |  |  |  | Abundance of target species | |  |  |  |  |
| Age | - | - | - | - | - | Age | - | - | - | - | - |
| Edu2 | -0.33 | 0.23 | -1.42 | 0.71 | 0.2 | Edu2 | -0.13 | 0.23 | -0.57 | - | 0.6 |
| Edu3 | -0.5 | 0.23 | -2.16 | 0.61 | 0.03 | Edu3 | -0.33 | 0.23 | -1.46 | - | 0.1 |
| Gender male | - | - | - | - | - | Gender male |  |  |  |  |  |
| Angl. Day | - | - | - | - | - | Angl. day | - | - | - | - | - |
| Angl. Club | - | - | - | - | - | Angl. club | - | - | - | - | - |
| Centrality | - | - | - | - | - | Centrality | - | - | - | - | - |
| Marine water | -0.27 | 0.22 | -1.19 | 0.77 | 0.2 | Marine water | -0.33 | 0.22 | -1.48 |  | 0.1 |
| Standing water | 0.23 | 0.16 | 1.45 | 1.26 | 0.2 | Standing water | ß.22 | 0.16 | 1.39 |  | 0.2 |
| Motive catch | - | - | - | - | - | Motive catch | - | - | - | - | - |
| Motive sport | - | - | - | - | - | Motive sport | - | - | - | - | - |
| Motive nature | - | - | - | - | - | Motive nature | - | - | - | - | - |
| Motive escape | - | - | - | - | - | Motive escape | - | - | - | - | - |
| NEP | - | - | - | - | - | NEP | - | - | - | - | - |
| SKILL | - | - | - | - | - | SKILL | - | - | - | - | - |
| Thr. coeff. 1-2 | -3.14 | 0.27 | -11.6 | 0.04 |  | Thr. coeff. 1-2 | -3.74 | 0.31 | -12.08 | 0.02 |  |
| Thr. coeff. 2-3 | -0.87 | 0.23 | -3.8 | 0.41 |  | Thr. coeff. 2-3 | -0.84 | 0.22 | -3.79 | 0.43 |  |
| Thr. coeff. 3-4 | 1.79 | 0.24 | 7.48 | 6.0 |  | Thr. coeff. 3-4 | 1.68 | 0.23 | 7.24 | 5.39 |  |
| Thr. coeff. 4-5 | 4.53 | 0.46 | 9.87 | 93.0 |  | Thr. coeff. 4-5 | 4.06 | 0.37 | 11.06 | 57.78 |  |
| Growth of target species | |  |  |  |  | Growth of target species | |  |  |  |  |
| Age | - | - | - | - | - | Age | - | - | - | - | - |
| Edu2 | - | - | - | - | - | Edu2 | - | - | - | - | - |
| Edu3 | - | - | - | - | - | Edu3 | - | - | - | - | - |
| Gender male | - | - | - | - | - | Gender male | 0.61 | 0.36 | 1.67 | 1.84 | 0.1 |
| Angl. Day | - | - | - | - | - | Angl. Day | - | - | - | - | - |
| Angl. Club | 0.31 | 0.17 | 1.9 | 1.37 | 0.06 | Angl. club | 0.25 | 0.16 | 1.54 | 1.29 | 0.1 |
| Centrality | - | - | - | - | - | Centrality | - | - | - | - | - |
| Marine water | -0.59 | 0.24 | -2.53 | 0.55 | 0.01 | Marine water | -0.48 | 0.23 | -2.07 | 0.62 | 0.04 |
| Standing water | 0.19 | 0.17 | 1.13 | 1.21 | 0.3 | Standing water | 0.34 | 0.17 | 2.1 | 1.41 | 0.04 |
| Motive catch | - | - | - | - | - | Motive catch | 0.13 | 0.08 | 1.67 | 1.14 | 0.1 |
| Motive sport | - | - | - | - | - | Motive sport | - | - | - | - | - |
| Motive nature | - | - | - | - | - | Motive nature | - | - | - | - | - |
| Motive escape | - | - | - | - | - | Motive escape | - | - | - | - | - |
| NEP | -0.37 | 0.16 | -2.26 | 0.7 | 0.02 | NEP | - | - | - | - | - |
| SKILL | -0.17 | 0.07 | 2.36 | 1.19 | 0.02 | SKILL | 0.14 | 0.07 | 1.87 | 1.15 | 0.06 |
| Thr. coeff. 1-2 | -3.82 | 0.71 | -5.42 | 0.02 |  | Thr. coeff. 1-2 | -2.4 | 0.47 | -5.1 | 0.09 |  |
| Thr. coeff. 2-3 | -1.72 | 0.68 | -2.52 | 0.18 |  | Thr. coeff. 2-3 | 0.19 | 0.43 | 0.46 | 1.22 |  |
| Thr. coeff. 3-4 | 1.43 | 0.68 | 2.1 | 4.18 |  | Thr. coeff. 3-4 | 2.98 | 0.44 | 6.73 | 19.69 |  |
| Thr. coeff. 4-5 | 4.27 | 0.79 | 5.42 | 71.65 |  | Thr. coeff. 4-5 | 6.38 | 0.63 | 10.2 | 589.0 |  |
| Fish kills |  |  |  |  |  | Fish kills |  |  |  |  |  |
| Age | -0.03 | 0.005 | -4.42 | 0.98 | < 0.0001 | Age | -0.02 | 0.005 | -4.1 | 0.98 | < 0.0001 |
| Edu2 | -0.26 | 0.26 | -1.02 | 0.77 | 0.3 | Edu2 | -0.11 | 0.25 | -0.42 | 0.9 | 0.7 |
| Edu3 | 0.18 | 0.25 | 0.72 | 1.2 | 0.5 | Edu3 | 0.4 | 0.25 | 1.6 | 1.49 | 0.1 |
| Gender male | - | - | - | - | - | Gender male | - | - | - | - | - |
| Angl. Day | - | - | - | - | - | Angl. Day | - | - | - | - | - |
| Angl. Club | - | - | - | - | - | Angl. club | - | - | - | - | - |
| Centrality | - | - | - | - | - | Centrality | -0.12 | 0.08 | -1.54 | 0.88 | 0.1 |
| Marine water | 0.005 | 0.24 | 0.02 | 1.01 | 0.9 | Marine water | - | - | - | - | . |
| Standing water | -0.33 | 0.17 | -1.91 | 0.72 | 0.06 | Standing water | - | - | - | - | . |
| Motive catch | 0.19 | 0.09 | 2.0 | 1.21 | 0.045 | Motive catch | . | . | . | . | . |
| Motive sport | 0.17 | 0.1 | -1.73 | 0.84 | 0.08 | Motive sport | - | - | - | - | . |
| Motive nature | 0.18 | 0.08 | 223 | 1.19 | 0.03 | Motive nature | - | - | - | - | - |
| Motive escape | - | - | - | - | - | Motive escape | - | - | - | - | - |
| NEP | 0.59 | 0.17 | 3.45 | 1.8 | 0.0006 | NEP | 0.5 | 0.16 | 3.1 | 1.65 | 0.002 |
| SKILL | -0.16 | 0.08 | 2.04 | 0.85 | 0.04 | SKILL | - | - | - | - | - |
| Thr. coeff. 1-2 | -4.35 | 0.83 | -5.23 | 0.01 |  | Thr. coeff. 1-2 | -4.29 | 0.84 | -5.12 | 0.01 |  |
| Thr. coeff. 2-3 | -2.38 | 0.77 | -3.07 | 0.09 |  | Thr. coeff. 2-3 | -1.97 | 0.75 | -2.64 | 0.14 |  |
| Thr. coeff. 3-4 | 1.27 | 0.77 | 1.66 | 3.57 |  | Thr. coeff. 3-4 | 1.36 | 0.74 | 1.84 | 3.89 |  |
| Thr. coeff. 4-5 | 3.82 | 0.79 | 4.81 | 45.63 |  | Thr. coeff. 4-5 | 4.11 | 0.77 | 5.35 | 60.65 |  |
| Phytoplankton blooms | |  |  |  |  | Phytoplankton blooms | |  |  |  |  |
| Age | - | - | - | - | - | Age | - | - | - | - | - |
| Edu2 | - | - | - | - | - | Edu2 | 0.28 | 0.23 | 1.19 | 1.32 | 0.2 |
| Edu3 | - | - | - | - | - | Edu3 | 0.53 | 0.23 | 2.34 | 1.71 | 0.02 |
| Gender male | - | - | - | - | - | Gender male | -0.82 | 0.37 | -2.23 | 0.44 | 003 |
| Angl. day | - | - | - | - | - | Angl. Day | - | - | - | - | - |
| Angl. club | 0.55 | 0.15 | 3.84 | 1.74 | 0.0001 | Angl. Club | 0.48 | 0.15 | 3.26 | 1.62 | 0.001 |
| Centrality | - | - | - | - | - | Centrality | - | - | - | - | - |
| Marine water | - | - | - | - | - | Marine water | - | - | - | - | - |
| Standing water | - | - | - | - | - | Standing water | - | - | - | - | - |
| Motive catch | - | - | - | - | - | Motive catch | - | - | - | - | - |
| Motive sport | - | - | - | - | - | Motive sport | - | - | - | - | - |
| Motive nature | - | - | - | - | - | Motive nature | - | - | - | - | - |
| Motive escape | - | - | - | - | - | Motive escape | - | - | - | - | - |
| NEP | 0.48 | 0.15 | 3.16 | 1.62 | 0.002 | NEP | 0.48 | 0.16 | 3.1 | 1.62 | 0.002 |
| SKILL | - | - | - | - | - | SKILL | - | - | - | - | - |
| Thr. coeff. 1-2 | -1.67 | 0.63 | -2.65 | 0.19 |  | Thr. coeff. 1-2 | -2.15 | 0.77 | -2.81 | 0.12 |  |
| Thr. coeff. 2-3 | -0.13 | 0.59 | -0.21 | 0.88 |  | Thr. coeff. 2-3 | -0.72 | 0.74 | -0.98 | 0.49 |  |
| Thr. coeff. 3-4 | 1.98 | 0.6 | 3.33 | 7.25 |  | Thr. coeff. 3-4 | 1.41 | 0.74 | 1.91 | 4.1 |  |
| Thr. coeff. 4-5 | 4.6 | 0.62 | 7.44 | 99.89 |  | Thr. coeff. 4-5 | 4.38 | 0.76 | 5.8 | 79.64 |  |
| Low water events |  |  |  |  |  | Low water events |  |  |  |  |  |
| Age | - | - | - | - | - | Age | - | - | - | - | - |
| Edu2 | -0.05 | 0.23 | -0.22 | 0.95 | 0.8 | Edu2 | 0.12 | 0.23 | 0.51 | 1.12 | 0.6 |
| Edu3 | 0.39 | 0.23 | 1.74 | 1.48 | 0.08 | Edu3 | 0.45 | 0.23 | 2.ß | 1.58 | 0.045 |
| Gender male | - | - | - | - | - | Gender male | - | - | - | - | - |
| Angl. day | - | - | - | - | - | Angl. Day | - | - | - | - | - |
| Angl. club | - | - | - | - | - | Angl. Club | - | - | - | - | - |
| Centrality | - | - | - | - | - | Centrality | - | - | - | - | - |
| Marine water | -1.19 | 0.22 | -5.49 | 0.31 | < 0.0001 | Marine waters | -1.22 | 0.22 | -5.56 | 0.29 | < 0.0001 |
| Standing water | -0.23 | 0.16 | -1.48 | 0.79 | 0.1 | Standing water | -0.12 | 0.16 | -0.76 | 0.89 | 0.4 |
| Motive catch | - | - | - | - | - | Motive catch | - | - | - | - | - |
| Motive sport | - | - | - | - | - | Motive sport | - | - | - | - | - |
| Motive nature | - | - | - | - | - | Motive nature | - | - | - | - | - |
| Motive escape | - | - | - | - | - | Motive escape | - | - | - | - | - |
| NEP | 0.37 | 0.15 | 2.41 | 1.45 | 0.02 | NEP | 0.5 | 0.16 | 3.23 | 1.65 | 0.001 |
| SKILL | 0.14 | 0.07 | 2.1 | 1.15 | 0.04 | SKILL | 0.14 | 0.07 | 2.02 | 1.15 | 0.04 |
| Thr. coeff. 1-2 | -2.55 | 0.71 | -3.58 | 0.08 |  | Thr. coeff. 1-2 | -2.15 | 0.73 | -2.95 | 0.12 |  |
| Thr. coeff. 2-3 | -0.64 | 0.66 | -0.98 | 0.53 |  | Thr. coeff. 2-3 | -0.2 | 0.66 | -0.31 | 0.82 |  |
| Thr. coeff. 3-4 | 1.83 | 0.66 | 2.78 | 6.23 |  | Thr. coeff. 3-4 | 2.53 | 0.67 | 3.8 | 12.54 |  |
| Thr. coeff. 4-5 | 4.69 | 0.68 | 6.87 | 109.2 |  | Thr. coeff. 4-5 | 5.1 | 0.69 | 7.4 | 164.1 |  |
| Flooding events |  |  |  |  |  | Flooding events |  |  |  |  |  |
| Age | -0.009 | 0.005 | -1.68 | 0.99 | 0.09 | Age | -0.01 | 0.005 | -2.3 | 0.99 | 0.02 |
| Edu2 | -0.06 | 0.24 | -0.27 | 0.94 | 0.8 | Edu2 | -0.38 | 0.23 | -1.6 | 0.69 | 0.1 |
| Edu3 | 0.26 | 0.23 | 1.11 | 1.29 | 0.3 | Edu3 | -0.07 | 0.23 | -0.3 | 0.93 | 0.8 |
| Gender male | - | - | - | - | - | Gender male | - | - | - | - | - |
| Angl. day | -0.006 | 0.002 | -2.88 | 1.0 | 0.004 | Angl. Day | -0.006 | 0.002 | 2.97 | 0.99 | 0.003 |
| Angl. club | -0.25 | 0.15 | -1.62 | 0.78 | 0.1 | Angl. Club | - | - | - | - | - |
| Centrality | - | - | - | - | - | Centrality | -0.14 | 0.09 | -1.63 | 0.87 | 0.1 |
| Marine water | - | - | - | - | - | Marine waters |  |  |  |  |  |
| Standing water | - | - | - | - | - | Standing water |  |  |  |  |  |
| Motive catch | - | - | - | - | - | Motive catch | - | - | - | - | - |
| Motive sport | - | - | - | - | - | Motive sport | - | - | - | - | - |
| Motive nature | - | - | - | - | - | Motive nature | - | - | - | - | - |
| Motive escape | - | - | - | - | - | Motive escape | - | - | - | - | - |
| NEP | 0.23 | 0.15 | 1.45 | 1.26 | 0.1 | NEP | 0.26 | 0.15 | 1.67 | 1.29 | 0.09 |
| SKILL | - | - | - | - | - | SKILL | - | - | - | - | - |
| Thr. coeff. 1-2 | -4.47 | 0.76 | -5.9 | 0.01 |  | Thr. coeff. 1-2 | -4.79 | 0.77 | -6.24 | 0.005 |  |
| Thr. coeff. 2-3 | -1.57 | 0.66 | -2.37 | 0.21 |  | Thr. coeff. 2-3 | -1.83 | 0.66 | -2.79 | 0.1 |  |
| Thr. coeff. 3-4 | 0.96 | 0.66 | 1.45 | 2.6 |  | Thr. coeff. 3-4 | 0.59 | 0.65 | 0.91 | 1.17 |  |
| Thr. coeff. 4-5 | 3.03 | 0.68 | 4.48 | 20.66 |  | Thr. coeff. 4-5 | 2.73 | 0.66 | 4.11 | 9.94 |  |
| Extreme weather events | | |  |  |  | Extreme weather events | |  |  |  |  |
| Age | - | - | - | - | - | Age | -0.01 | 0.006 | -2.09 | 0.99 | 0.04 |
| Edu2 | -0.27 | 0.24 | -1.1 | 0.77 | 0.3 | Edu2 | -0.33 | 0.25 | -1.32 | 0.72 | 0.2 |
| Edu3 | 0.36 | 0.24 | 1.53 | 1.44 | 0.1 | Edu3 | 0.37 | 0.25 | 1.5 | 1.45 | 0.1 |
| Gender male | - | - | - | - | - | Gender male | - | - | - | - | - |
| Angl. day | -0.004 | 0.002 | -1.88 | 1.0 | 0.06 | Angl. Day | - | - | - | - | - |
| Angl. club | - | - | - | - | - | Angl. Club | - | - | - | - | - |
| Centrality | - | - | - | - | - | Centrality | -0.2 | 0.09 | -2.33 | 0.82 | 0.02 |
| Marine water | -0.53 | 0.23 | -2.35 | 0.59 | 0.02 | Marine waters | -0.54 | 0.23 | -2.34 | 0.58 | 0.02 |
| Standing water | -0.12 | 0.17 | -0.73 | 0.88 | 0.5 | Standing water | -0.15 | 0.17 | .0.88 | 0.86 | 0.4 |
| Motive catch | - | - | - | - | - | Motive catch | 0.19 | 0.09 | 2.18 | 1.21 | 0.02 |
| Motive sport | - | - | - | - | - | Motive sport | -0.22 | 0.09 | -2.3 | 0.81 | 0.02 |
| Motive nature | - | - | - | - | - | Motive nature | - | - | - | - | - |
| Motive escape | - | - | - | - | - | Motive escape | - | - | - | - | - |
| NEP | 0.76 | 0.16 | 4.64 | 2.13 | < 0.0001 | NEP | 0.87 | 0.17 | 5.19 | 2.38 | < 0.0001 |
| SKILL | - | - | - | - | - | SKILL | - | - | - | - | - |
| Thr. coeff. 1-2 | -2.21 | 0.76 | -2.91 | 0.11 |  | Thr. coeff. 1-2 | -4.08 | 1.03 | -3.96 | 0.02 |  |
| Thr. coeff. 2-3 | -1.04 | 0.68 | -1.52 | 0.36 |  | Thr. coeff. 2-3 | -2.27 | 0.8 | -2.83 | 0.1 |  |
| Thr. coeff. 3-4 | 1.85 | 0.65 | 2.82 | 6.33 |  | Thr. coeff. 3-4 | 0.99 | 0.76 | 1.31 | 2.71 |  |
| Thr. coeff. 4-5 | 5.03 | 0.68 | 7.36 | 153.2 |  | Thr. coeff. 4-5 | 4.28 | 0.78 | 5.47 | 71.97 |  |

Suppl. Tab. S5: Model fits of the final ordinal regression models regarding observed and assumed effects of climate change on fish species and aquatic ecosystems.

| Statistics | To what extent have climate changes in recent years already influenced the following aspects of your main fishing water: | To what extent will climate change influence the following aspects of your main fishing water in the future: |
| --- | --- | --- |
|  | **Spread of new fish species** | **Spread of new fish species** |
| Lipsitz test | LR statistic = 9.5, df = 9, *p* = 0.4 | LR statistic = 10.5, df = 9, *p* = 0.3 |
| Hosmer and Lemeshow test | *Chi*² = 25.6, df = 15, *p* = 0.04 | *Chi*²= 10.6, df = 15, *p* = 0.78 |
| Nagelkerke r² | 0.06 | 0.05 |
| Likelihood test model vs. zero model | Dfdiff = -8, LogLik.diff = -20.5  *Chi*² = 41.0, *p* < 0.0001 | Dfdiff = -6, LogLik.diff = -15.6  *Chi*² = 31.3, *p* < 0.0001 |
| Brant test | Proportional odds assumption holds | Proportional odds assumption holds |
|  | **Loss of fish species** | **Loss of fish species** |
| Lipsitz test | LR statistic = 10.3, df = 9, *p* = 0.3 | LR statistic = 10.6, df = 9, *p* = 0.3 |
| Hosmer and Lemeshow test | *Chi*² = 20.5, df = 15, *p* = 0.2 | *Chi*² = 22.4, df = 15, *p* = 0.1 |
| Nagelkerke r² | 0.05 | 0.06 |
| Likelihood test model vs. zero model | Df.diff = -7, LogLik.diff = -16.0  *Chi*² = 32.0, *p* = 0.0001 | Df.diff = -4, LogLik.diff = -19.5  *Chi*² = 38.9, *p* < 0.0001 |
| Brant test | Proportional odds assumption holds | Proportional odds assumption holds |
|  | **Reproduction of target species** | **Reproduction of target species** |
| Lipsitz test | LR statistic = 7.9, df = 9, *p* = 0.5 | LR statistic = 5.2, df = 9, *p* = 0.8 |
| Hosmer and Lemeshow test | *Chi*² = 19.1, df = 15, *p* = 0.2 | *Chi*² = 19.5, df = 15, *p* = 0.2 |
| Nagelkerke r² | 0.05 | 0.03 |
| Likelihood test model vs. zero model | Df.diff = -4, LogLik.diff = -16.7  *Chi*² = 33.4, *p* < 0.0001 | Dfdiff = -6, LogLik.diff = -9.4  *Chi*² = 18.8, *p* = 0.005 |
| Brant test | Proportional odds assumption holds | Proportional odds assumption holds |
|  | **Abundance of target species** | **Abundance of target species** |
| Lipsitz test | LR statistic = 2.5, df = 9, *p* = 0.9 | LR statistic = 7.3, df = 9, *p* = 0.6 |
| Hosmer and Lemeshow test | *Chi*² = 27.7, df = 15, *p* = 0.02 | *Chi*² = 27.1, df = 15, *p* = 0.03 |
| Nagelkerke r² | 0.02 | 0.02 |
| Likelihood test model vs. zero model | Df.diff = -4, LogLik.diff = -6.0  *Chi*² = 12.0, *p* = 0.02 | Df.diff = -4, LogLik.diff = -5.4,  *Chi*² = 10.9, *p* = 0.03 |
| Brant test | Proportional odds assumption holds | Proportional odds assumption holds |
|  | **Growth of target species** | **Growth of target species** |
| Lipsitz test | LR statistic = 9.8, df = 9, *p* = 0.4 | LR statistic = 6.6, df = 9, *p* = 0.7 |
| Hosmer and Lemeshow test | *Chi*² = 9.3, df = 15, *p* = 0.9 | *Chi*² = 36.7, df = 15, *p* = 0.001 |
| Nagelkerke r² | 0.05 | 0.05 |
| Likelihood test model vs. zero model | Df.diff = -5, LogLik.diff = -17  *Chi*² = 34.0, *p* = 0.0001 | Df.diff = -6, LogLik.diff = -17.6  *Chi*² = 35.3, *p* < 0.0001 |
| Brant test | Proportional odds assumption holds | Proportional odds assumption holds |
|  | **Fish kills** | **Fish kills** |
| Lipsitz test | LR statistic = 5.8, df = 9, *p* = 0.8 | LR statistic = 10.6, df = 9, *p* = 0.3 |
| Hosmer and Lemeshow test | *Chi*² = 11.7, df = 15, *p* = 0.7 | *Chi*² = 18.0, df = 15, *p* = 0.3 |
| Nagelkerke r² | 0.08 | 0.06 |
| Likelihood test model vs. zero model | Df.diff= -10, LogLik.diff = -26.5  *Chi*² = 52.9, *p* < 0.0001 | Df.diff = -5, LogLik.diff = -20.1,  *Chi*² = 40.3, *p* < 0.0001 |
| Brant test | Proportional odds assumption holds | Proportional odds assumption holds |
|  | **Phytoplankton blooms** | **Phytoplankton blooms** |
| Lipsitz test | LR statistic = 9.0, df = 9, *p* = 0.74 | LR statistic = 5.4, df = 9, *p* = 0.8 |
| Hosmer and Lemeshow test | *Chi*² = 12.7, df = 15, *p* = 0.6 | Chi² = 7.9, df = 15, *p* = 0.9 |
| Nagelkerke r² | 0.04 | 0.05 |
| Likelihood test model vs. zero model | Df.diff = -2, LogLik.diff = -11.9  *Chi*² = 23.8, *p* < 0.0001 | Df.diff = -5, LogLik.diff = -15.0  *Chi*² = 29.9 *p* = 0.0001 |
| Brant test | Proportional odds assumption holds | Proportional odds assumption holds |
|  | **Low water events** | **Low water events** |
| Lipsitz test | LR statistic = 9.9, df = 9, *p* = 0.4 | LR statistic = 6.1, df = 9, *p* = 0.7 |
| Hosmer and Lemeshow test | *Chi*² = 38.9, df = 15, *p* = 0.0007 | *Chi*² = 24.8, df = 15, *p* = 0.053 |
| Nagelkerke r² | 0.08 | 0.08 |
| Likelihood test model vs. zero model | Df.diff = -6, LogLik.diff = -26.6  *Chi*² = 53.3, *p* < 0.0001 | Df.diff = -6, LogLik.diff = -28.3  *Chi*² = 56.7, *p* < 0.0001 |
| Brant test | Proportional odds assumption holds | Proportional odds assumption holds |
|  | **Flooding** | **Flooding** |
| Lipsitz test | LR statistic = 2.8, df = 9, *p* = 1.0 | LR statistic = 12.5, df = 9, *p* = 0.2 |
| Hosmer and Lemeshow test | *Chi*² = 12.5, df = 15, *p* = 0.6 | *Chi*² = 12.3, df = 15, *p* = 0.7 |
| Nagelkerke r² | 0.04 | 0.05 |
| Likelihood test model vs. zero model | Df.diff = -6, LogLik.diff = -12.2  *Chi*² = 24.5, *p* = 0.0004 | Df.diff = -6, LogLik.diff = -15.85  *Chi*² = 31.0, *p* < 0.0001 |
| Brant test | Proportional odds assumption holds | Proportional odds assumption holds |
|  | **Extreme weather events 20** | **Extreme weather events** |
| Lipsitz test | LR statistic = 4.7, df = 9, *p* = 0.9 | LR statistic = 13.1, df = 9, *p* = 0.2 |
| Hosmer and Lemeshow test | *Chi*² = 14.9, df = 15, *p* = 0.5 | *Chi*² = 25.7, df = 15, *p* = 0.04 |
| Nagelkerke r² | 0.07 | 0.1 |
| Likelihood test model vs. zero model | Df.diff = -6, LogLik.diff = -22.9  *Chi*² = 45.8, *p* < 0.0001 | Df.diff = -8, LogLik.diff = -35.2  *Chi*² = 70.4, *p* < 0.0001 |
| Brant test | Proportional odds assumption holds | Proportional odds assumption holds |


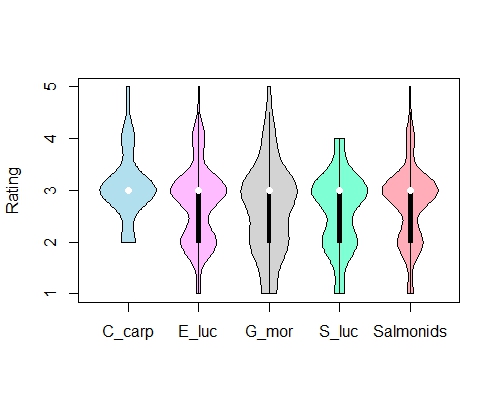


Suppl. Fig. S1: Violin plot showing the distribution of the ratings of the statement „To what extent have climate changes in recent years already influenced the abundance of your target fish species?” by anglers targeting carp (C_carp), pike (E_luc), pikeperch (S_luc), and salmonids, mainly trout (*S. trutta*). KW test: *chi*² = 20.31, *p* = 0.0004; Wilcoxon pairwise test with FDR adjustment: carp/pike: *p* = 0.003, carp/cod: 0.002, carp/pikeperch: *p* = 0.0004, carp/salmonids: *p* = 0.003, pike/cod: *p* = 0.4, pike/pikeperch: *p* = 0.4, pike/salmonids: *p* = 0.9, cod/pikeperch: *p* = 1.0, cod/salmonids: *p* = 0.4, pikeperch/salmonids: *p* = 0.3.


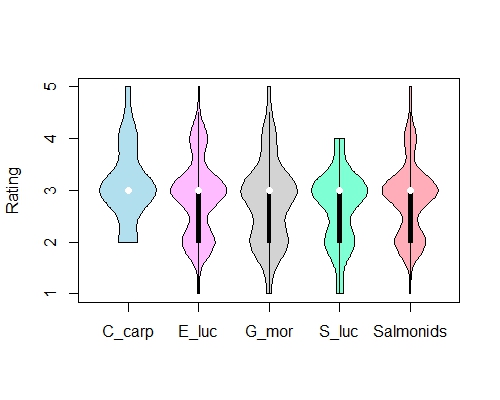


Suppl. Fig. S2: Violin plot showing the distribution of the ratings of the statement „To what extent will climate changes influence the abundance of your target fish species?” by anglers targeting carp (C_carp), pike (E_luc), pikeperch (S_luc), and salmonids, mainly trout (*S. trutta*). KW test: *chi*² = 15.73, *p* = 0.003; Wilcoxon pairwise test with FDR adjustment: carp/pike: *p* = 0.03, carp/cod: 0.01, carp/pikeperch: *p* = 0.002, carp/salmonids: *p* = 0.01, pike/cod: *p* = 0.6, pike/pikeperch: *p* = 0.2, pike/salmonids: *p* = 0.8, cod/pikeperch: *p* = 0.6, cod/salmonids: *p* = 0.6, pikeperch/salmonids: *p* = 0.2.


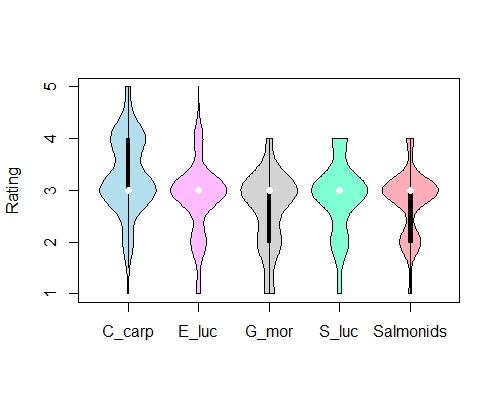


Suppl. Fig. S3: Violin plot showing the distribution of the ratings of the statement “To what extent have climate changes in recent years already influenced the growth of your target fish species?” by anglers targeting carp (C_carp), pike (E_luc), pikeperch (S_luc), and salmonids, mainly trout (*S. trutta*). KW test: chi² = 44.85, *p* < 0.0001; Wilcoxon pairwise test with FDR adjustment: carp/pike: *p* < 0.0001, carp/cod: *p* < 0.0001, carp/pikeperch: *p* = 0.0003, carp/salmonids: *p* < 0.0001, pike/cod: *p* = 0.04, pike/pikeperch: *p* = 0.5, pike/salmonids: *p* = 0.4, cod/pikeperch: *p* = 0.02, cod/salmonids: *p* = 0.1, pikeperch/salmonids: *p* = 0.2.


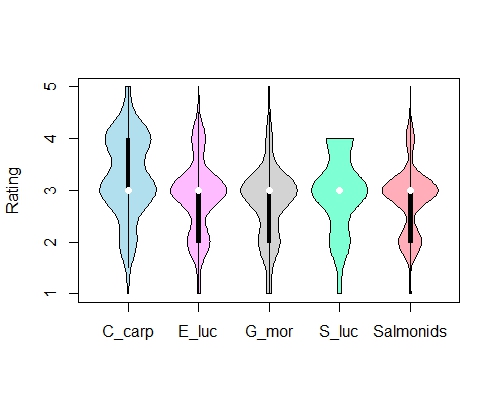


Suppl. Fig. S4: Violin plot showing the distribution of the ratings of the statement “To what extent will climate changes influence the growth of your target fish species?” by anglers targeting carp (C_carp), pike (E_luc), pikeperch (S_luc), and salmonids, mainly trout (*S. trutta*). KW test: *chi*² = 33.14, *p* < 0.0001; Wilcoxon pairwise test with FDR adjustment: carp/pike: *p* = 0.0001, carp/cod: *p* < 0.00001, carp/pikeperch: *p* = 0.03, carp/salmonids: *p* < 0.00001, pike/cod: *p* = 0.3, pike/pikeperch: *p* = 0.2, pike/salmonids: *p* = 0.5, cod/pikeperch: *p* = 0.03, cod/salmonids: *p* = 0.5, pikeperch/salmonids: *p* = 0.03.

.


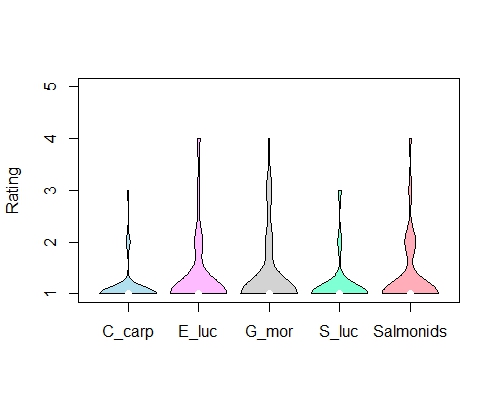


Suppl. Fig. S5: Violin plot showing the distribution of the ratings of the statement „I will stop angling due to climatic changes” by anglers targeting carp (C_carp), pike (E_luc), cod (G_mor), pikeperch (S_luc), and salmonids, mainly trout (*S. trutta*). KW test: *chi*² = 12.14, *p* = 0.02; Wilcoxon pairwise test with FDR adjustment: carp/pike: *p* = 0.06, carp/cod: 0.06, carp/pikeperch: *p* = 0.6, carp/salmonids: *p* = 0.04, pike/cod: *p* = 0.9, pike/pikeperch: *p* = 0.1, pike/salmonids: *p* = 0.7, cod/pikeperch: *p* = 0.1, cod/salmonids: *p* = 0.9, pikeperch/salmonids: *p* = 0.06.
